# Repair versus Debridement for Acetabular Labral Tears—A Systematic Review

**DOI:** 10.1016/j.asmr.2021.06.008

**Published:** 2021-08-18

**Authors:** Eoghan T. Hurley, Andrew J. Hughes, M. Shazil Jamal, Edward S. Mojica, David A. Bloom, Thomas Youm, Tom McCarthy

**Affiliations:** a New York University Langone Health, Department of Orthopaedic Surgery, New York, New York, U.S.A.; b Sports Surgery Clinic, Dublin, Ireland

## Abstract

**Purpose:**

The purpose of this study was to systematically review the evidence in the literature to ascertain whether acetabular labral repair (ALR) or debridement (ALD) resulted in superior patient outcomes.

**Methods:**

The systematic review was conducted in accordance with the Preferred Reporting Items for Systematic Reviews and Meta-Analyses guidelines. Peer-reviewed studies comparing ALR and ALD published in English with full text available were included. Patients undergoing both open and arthroscopic surgery in randomized controlled trials, prospective cohort studies, retrospective cohort studies, and case-control studies were included. Studies were quantified for methodological quality using the MINORS criteria. Clinical outcomes were compared, with qualitative analysis, and quantitative analyses were performed using GraphPad Prism version 7. A *P* value <.05 was considered to be statistically significant.

**Results:**

There were 8 studies included (level of evidence [LOE] I = 1; LOE II = 2; LOE III = 5). The 7 studies compared 364 patients (369 hips) with ALR to 318 patients (329 hips) with ALD, with a mean follow-up time ranging between 32-120 months. Five studies found significantly improved patient reported outcomes with ALR (Harris Hip Score, Merle d’Aubigné, Pain, SF-12). Several studies compared the outcomes after ALR and ALD and found statistical significance in all investigated metrics in favor of ALR. One study found a significant improvement in abduction but no other study found any difference in range of motion. No study found any difference in complication rate, revision rate or conversion to total hip arthroplasty. Although, 2 studies found ALR reduced the rate of osteoarthritic progression.

**Conclusion:**

Current literature suggests that acetabular labral repair may result in superior patient reported outcomes. However, there appears to be no significant difference in the rate of progression to total hip arthroplasty at up to 10-year follow-up.

**Level of Evidence:**

Level III, systematic review of Level I, II, and III studies.

Femoroacetabular impingement (FAI) syndrome can cause pain, dysfunction, and early arthritic progression. FAI syndrome occurs because of morphological abnormality of the bone in either the acetabular rim (pincer morphology) or femoral neck (cam morphology). Labral tears are common alongside FAI and occur in up to 55% of patients with mechanical symptoms, as a result of repetitive abnormal contact between the bone and labrum.^1^ Hip arthroscopy, while technically challenging, is increasingly being performed to treat FAI syndrome and associated pathologies.^2^, ^3^, ^4^ Advantages over the open approach include faster rehabilitation and avoidance of a trochanteric osteotomy.^4^^,^^5^

In the recent past, acetabular labral debridement (ALD) has been used to treat labral pathology and has been shown to reduce symptoms associated with FAI.^4^^,^^5^ However, the labrum has been shown to play an important role in joint stability, increasing the acetabular joint area and reducing forces transmitted through the articular cartilage; thus acetabular labral repair (ALR) may be advantageous in preserving the hip joint and reduce the rate of osteoarthritic progression.^6^, ^7^, ^8^, ^9^

There is limited evidence to suggest how such patients should be managed, nd whether labral tears should be repaired, because to our knowledge only 1 randomized controlled trial comparing ALR to ALD exists. Additional comparative studies have been published in recent years, warranting an updated systematic review. The purpose of this study was to systematically review the evidence in the literature to ascertain whether ALR or ALD resulted in superior patient outcomes. Our hypothesis was that ALR would result in superior patient reported outcomes, with lower progression rates to total hip arthroplasty.

## Methods

### Search Strategy and Study Selection

The literature search was conducted by two independent reviewers based on the Preferred Reporting Items for Systematic Reviews and Meta-Analyses guidelines.^10^ The MEDLINE, EMBASE, and The Cochrane Library databases were queried in April 2020 with the following search terms: (femoroacetabular or FAI or hip or coxa or acetabulofemoral joint) and (impingement) and (repair or refixation or preservation or reattachment or debridement or resection). The search results were reviewed independently and compared, with a senior author arbitrating in the instance of disagreement. The title and abstract of all search results were reviewed, and the full text of potentially eligible studies was evaluated. The reference lists of the included studies and literature reviews were manually screened for additional articles meeting the inclusion criteria that were not identified during the initial search. There was no time limit with respect to publication date.

### Eligibility Criteria

The inclusion criteria for this analysis were as follows: (1) studies comparing ALR and ALD, (2) published in a peer-reviewed journal, (3) published in English, and (4) full text of studies available. Study designs including randomized controlled trials, prospective cohort studies, retrospective cohort studies, and case control studies were considered for inclusion. The exclusion criteria were as follows: (1) case series, (2) review studies, (3) cadaver studies, (4) biomechanical studies, or (5) conference abstract only.

### Data Extraction/Analysis

The relevant study characteristics including study design, level of evidence, methodological quality of evidence, patient population, outcome measures, and follow-up time points were collected by 2 independent reviewers using a predetermined data sheet. The results from each reviewer were compared. The MINORS (Methodological Index for Non-Randomised Studies) was used to evaluate the potential assessed risk of bias for each included study.^11^ The items were scored 0 if not reported, 1 if reported inadequately, and 2 if reported adequately; the global ideal score was 16 for noncomparative and 24 for comparative studies.

### Statistical Analysis

Statistical analysis was performed using GraphPad Prism version 7. Qualitative analysis was performed for each study, and quantitative analysis was performed across all groups. Graphical representation of the comparative studies was performed using Review Manager ([RevMan; Macintosh]. Version 5.3. Copenhagen: The Nordic Cochrane Centre, The Cochrane Collaboration, 2014.).

## Results

### Literature Search

**Fig 1:**
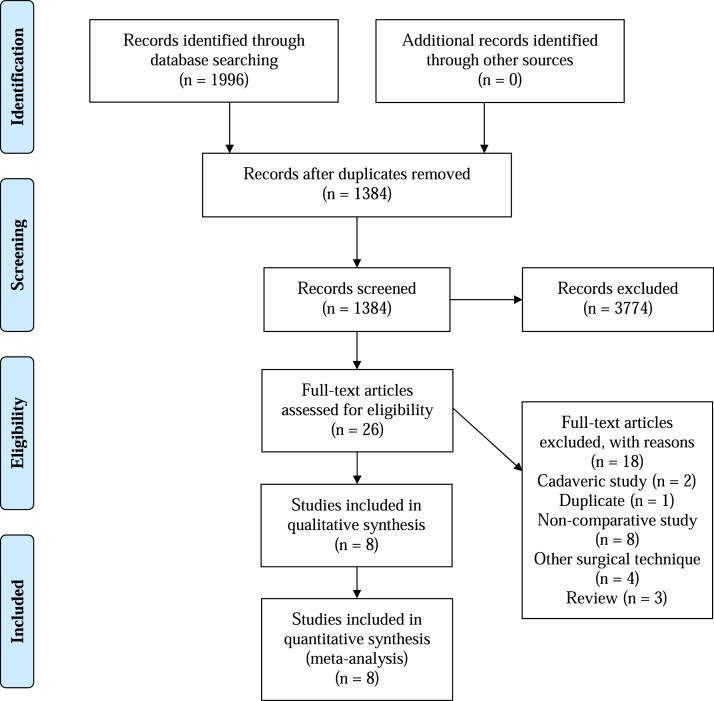
Preferred Reporting Items for Systematic Reviews and Meta-Analyses Study Selection Flow Diagram.

The initial literature search resulted in 1996 total studies. Once duplicates were removed and the articles were screened for inclusion and exclusion criteria, 1384 studies were included, and full texts were assessed for eligibility. Eight clinical studies with 682 patients (698 hips) were included in this review (Fig 1).

### Study Characteristics

Eight studies were included (level of evidence [LOE] I; 1, LOE II; 2, LOE III; 5). The 8 studies compared 364 patients (369 hips) with ALR to 318 patients (329 hips) with ALD, with a mean follow-up time ranging between 32 to 120 months.^12^, ^13^, ^14^, ^15^, ^16^, ^17^, ^18^, ^19^ Six studies used arthroscopic techniques, and 2 studies used an open approach to the hip. There was no significant difference between the cohorts in age, gender, concomitant cartilage injuries, or other reported baseline in the included studies. The study characteristics & patient demographics are reported in Table 1, and the MINORS score are shown in Table 2.

**Table 1:** Study Characteristics

Author	LOE	MINORS	O/A	ALD N (Hips)	ALR N (Hips)	Age (yrs.)	Follow up (mo.)
Anwander et al.^12^	III	19	O	17 (21)	28 (30)	29 (17–40)	156 (144-168)
Cetinkaya et al.^13^	II	18	A	34 (39)	33 (34)	36.5 (18-61)	46 (29-65)
Chen et al.^14^	III	20	A	69	69	44 (15-75)	67 (60-92)
Espinosa et al.^15^	III	18	O	25	35	30 (20-40)	N/R
Krych et al.^16^	I	19	A	18	18	39 (19-59)	32 (12-48)
Larson et al.^17^	II	18	A	42 (44)	48 (50)	30 (16-57)	42 (24-72)
Menge et al.^18^	III	21	A	71	74	41 (N/R)	>120
Schilders et al.^19^	III	21	A	32	69	37 (15-71)	29 (24-48)

LOE, level of evidence; O/A, open/arthroscopic; ALD, debridement; ALR, repair; N, number; yrs, years; mo., months.

**Table 2:** MINORS Score

Study Items	Anwander et al.^12^	Cetinkaya et al.^13^	Chen et al.^14^	Espinosa et al.^15^	Krych et al.^16^	Larson et al.^17^	Menge et al.^18^	Schilders et al.^19^
Clearly stated aim	2	1	2	2	2	2	2	2
Inclusion of consecutive patients	2	2	2	2	2	2	2	2
Prospective data collection	1	1	2	1	1	1	2	2
Endpoints appropriate to study aims	2	2	2	2	2	2	2	2
Unbiased assessment of study endpoint	2	1	1	1	1	1	1	1
F/u period appropriate to aim of study	2	2	2	2	2	2	2	2
<5% lost to follow-up	2	2	1	1	2	1	1	2
Prospective calculation of study size	0	0	1	0	0	0	1	0
Adequate control group	1	2	2	2	2	2	2	2
Contemporary groups	1	2	1	2	2	1	2	2
Baseline equivalence	2	1	2	1	1	2	2	2
Adequate statistical analyses	2	2	2	2	2	2	2	2
Total score	19/24	18/24	20/24	18/24	19/24	18/24	21/24	21/24

### Functional Outcomes

**Fig 2:**
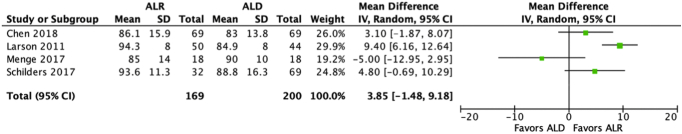
Forest Plot of the Harris Hip Score.

**Fig 3:**
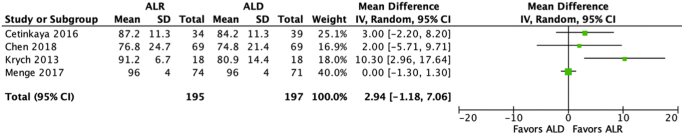
Forest Plot of the Hip Outcome Score.

**Fig 4:**
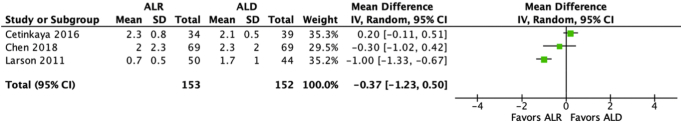
Forest Plot of the VAS Score.

All 8 studies compared functional outcomes between patients treated with ALR and ALD (Table 3). Three studies compared patient satisfaction scores, with one study finding a significant difference in favor of ALR. Four studies compared modified Harris Hip Scores, with one study finding a significant difference in favor of ALR, as shown in Fig 2. Four studies compared Hip Outcome Scores, with one study finding a significant difference in favor of ALR as shown in Fig 3. Three studies compared SF-12 scores, with one study finding a significant difference in favor of ALR. Five studies (3 using VAS score, and 2 using the Merle d'Aubigné compared pain scores, with two studies finding a significant difference in favor of ALR, with the VAS score shown in Fig 4. Overall, 5 studies found significantly improved patient reported outcomes with ALR.

**Table 3:** Functional Outcomes

Study	HOS (ALR vs ALD)	mHHS (ALR vs ALD)	SF-12 Score (ALR vs ALD)	SF-12 MCS (ALR vs ALD)	SF-12 PCS (ALR vs ALD)	Patient Satisfaction (ALR vs ALD)	VAS (ALR vs ALD)	Merle d'Aubigné (ALR vs ALD)
Anwander et al.^12^								5∗ (3-6) vs 3.9 (0-6)
Cetinkaya et al.^13^	87.2 (50-99) vs 84.2 (50-98)						2.3 (0-3) vs 2.1 (1-3)	
Chen et al.^14^	76.8 ± 24.7 vs 74.8 ± 21.4	86.1 ± 14.9 vs 83.0 ± 13.8		55.1 ± 7.3 vs 57.4 ± 5.3	48.9 ± 9.5 vs 48.7 ± 7.6	8.3 ± 2.2 vs 8.0 ± 2.0	2.0 ± 2.3 vs 2.3 ± 2.0	
Espinosa et al. ^15^								5.6∗ (1-6) vs 4.0 (0-6)
Krych et al.^16^	91.2∗ (73-100) vs 80.9 (43-100)					RNR∗		
Larson et al.^17^		94.3∗ vs 84.9	89.8∗ vs 82.2				0.7 vs 1.7	
Menge et al.^18^	96 (88-100) vs 96 (89-100)	85 (63-99) vs 90 (85-100)			56 (47-58) vs 56 (51-58)	10 vs 10		
Schilders et al.^19^		93.6∗ (55-100) vs 88.8 (35-100)						

Range or Standard Deviation was not reported for Larson et al.^17^

HOS; hip outcome score, mHHS; modified Harris Hip Score, SF-12; short form, MCS; mental score, PCS; physical score, VAS; visual analogue scale, ALD; debridement, ALR; repair.

∗ Denotes Statistical significance in favor of ALR.

### Range of Motion

Two studies compared range of motion between the two cohorts (Table 4), and one study found a significant difference in favor of ALR for abduction, but neither found a difference in flexion, extension, external rotation, internal rotation, or adduction.

**Table 4:** Range of Motion

Study	Flexion (ALR vs ALD)	Extension (ALR vs ALD)	External Rotation (ALR vs ALD)	Internal Rotation (ALR vs ALD)	Abduction (ALR vs ALD)	Adduction (ALR vs ALD)
Anwander et al.^12^	102 (70-130) vs 99 (70-120)	5 (0-10) vs 5 (0-10)	36 (10-75) vs 39 (5-80)	15 (0-45) vs 8 (0-45)	45∗ (30-70) vs 38 (25-45)	22 (15-30) vs 20 (0-40)
Espinosa et al.^15^	105 vs 96		49 vs 35		56 vs 47	

ALD, debridement; ALR. repair.

∗ Denotes statistical significance in favor of ALR.

### Revisions

**Fig 5:**
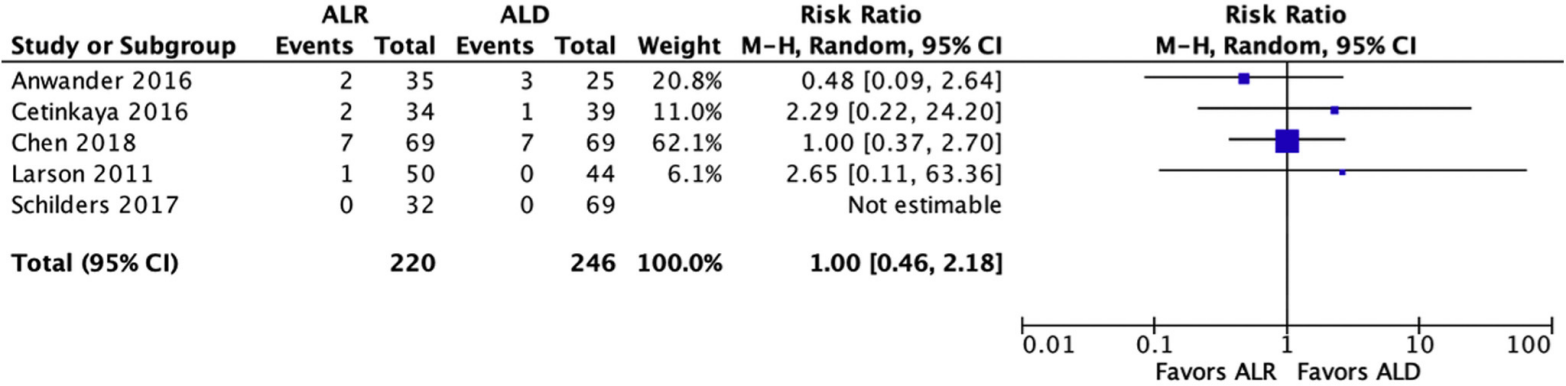
Forest Plot of the rate of conversion to total hip arthroplasty.

**Fig 6:**
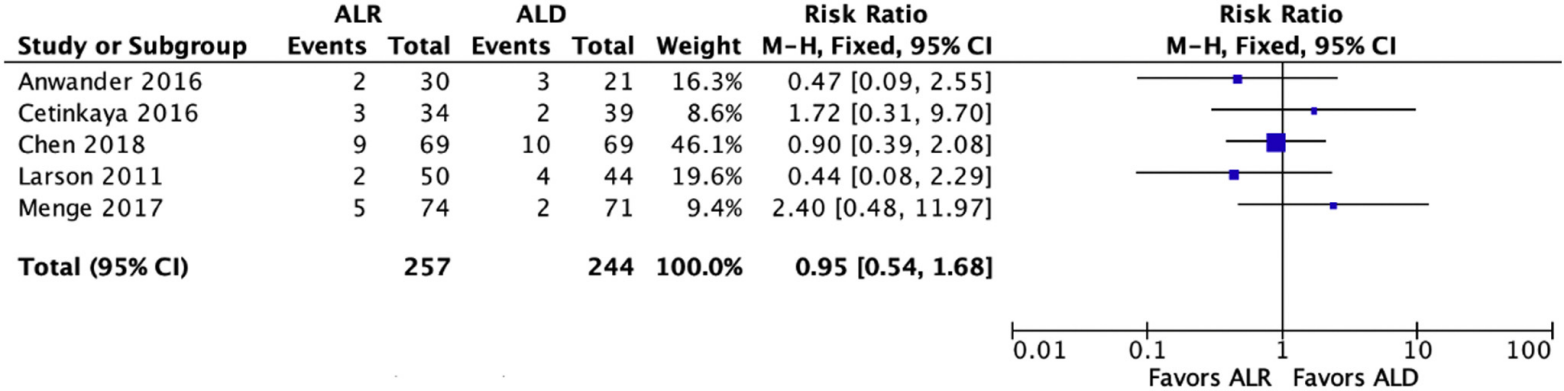
Forest Plot of the revision rate.

Six studies compared the rate of conversion to total hip arthroplasty (Table 5), with no study finding a significant difference in favor of either procedure, as shown in Fig 5. Additionally, 5 studies compared the rate of total revisions, with no study finding a significant difference in favor of either procedure, as shown in Fig 6.

**Table 5:** Revisions

Study	THR (ALR vs ALD)	Revision (ALR vs ALD)
Anwander et al.^12^	6% vs 12%	6% vs 12%
Cetinkaya et al.^13^	6% vs 3%	8.8% vs 6.1%
Chen et al.^14^	10.1% vs 10.1%	13.0% vs 14.4%
Larson et al.^17^	2.5% vs 0%	5.0% vs 9.1%
Menge et al.^18^	34%U	6.6% vs 2.7%
Schilders et al. 2017^19^	0% vs 0%	

THR, total hip replacement; U, results undifferentiated between groups; ALD, debridement; ALR, repair.

### Complications and Osteoarthritic Progression

Four studies compared complications between patients treated with ALR and ALD, with no study finding a significant difference in favor of either procedure (Table 6). However, 2 studies found the rate of overall osteoarthritic progression was significantly improved with ALR.

**Table 6:** Complications

Study	Complications (ALR vs ALD)	Arthritis (ALR vs ALD)
Anwander et al.^12^		78%∗ vs 46%
Cetinkaya et al.^13^	6U - All nerve palsies (2 femoral, 2 obturator and 2 pudendal)	
Chen et al.^14^	1.4% (1 nerve palsy – Perineal) vs 5.8% (2 nerve palsy – Sciatic and lateral femoral cutaneous, 1 infection, 1 pulmonary embolism)	
Espinosa et al.^15^		RNR∗
Schilders et al.^19^	0% vs 6% (3 heterotopic ossification)	

U, results undifferentiated between groups; ALD, debridement; ALR, repair; RNR, rate not reported.

∗ Denotes Statistical significance in favor of AL.

## Discussion

The primary finding of this study is that ALR may result in superior patient-reported outcomes relative to ALD. This is best supported by the fact that 5 of 8 included studies reported that ALR resulted in superior outcomes with statistically significant differences when compared to ALD. To further bolster this argument, there were no statistically significant differences in the rate of total complications, the rate of total revisions, and the rate of conversion to total hip arthroplasty between these 2 procedures. Additionally, it should be noted that 2 of the included studies demonstrated that the rate of osteoarthritis progression was significantly improved with ALR relative to ALD.

The results of this review supports the results of several previous biomechanical studies, which demonstrated that ALR restores the suction seal of the normal labrum, reduces femoral head translation, and reduces acetabular contact stress.^20^^,^^21^ The acetabular labrum is generally thought to provide normal hip function, in part, by acting as a stabilizer to distracting forces via the “suction effect” of the hip fluid seal. Research by Nepple et al.^20^ and Philippon et al.^21^ has demonstrated that the labrum is a significantly greater stabilizer than the capsule and accounts for 70% to 77% of distraction stabilization in the hip joint. Although both labral repair and debridement are important procedures for all hip arthroscopists, the significance of repair cannot be understated.

A recent survey-based study by Herickhoff et al.^22^ demonstrated that 4 variables known before surgery were identified as being important to the decision-making of the majority of hip arthroscopists. First, 70% of surveyed surgeons felt that magnetic resonance imaging was an important deciding factor—specifically, the arthritic status of the joint.^22^ Second, 63% of those surveyed favored labral repair in patients 52 ± 9.08 years and younger, although they favored debridement in patients 54 ± 6.41 years and older.^22^ Additionally, 57% favored repair in more active patients relative to less active ones, and 53% favored debridement for patients with Tonnis grade 2 or 3 on x-ray imaging.^22^

These survey results are especially interesting when considering the results of a recent study by Chen et al.,^14^ which attempted to prove that labral debridement, when used with narrow indications in select patients, had comparable 5-year outcomes to labral repair. Included patients had grade <4 Outerbridge chondral damage, preoperative Tonnis grade <2, no history of prior hip conditions or dysplasia, and no prior hip surgery. These patients were also required to have a stable labral base, at least 4 mm of the labral width, small focal tears with minimal intrasubstance involvement and enough stable labral tissue preserved to maintain the suction-seal function. Of note, the study demonstrated no statistically significant differences with respect to final patient-reported outcome scores between these matched groups with a similar rate of complications.

Byrd and Jones.^23^ described that arthroscopic selective debridement could result in favorable long-term outcome supported by a 10-year follow-up in 50 patients who underwent selective labral debridement. However, it is important to note that patients among the Byrd cohort fared worse with greater arthritis of the hip joint. In cases where the extent or pattern of labral damage was not amenable to repair, debridement of the damaged labrum may not be the preferred option, as labral reconstruction with an autograft or allograft may be a superiorly effective treatment option.^24^ In general, it appears that there is a general trend toward the preservation of the labrum with techniques such as labral repair or reconstruction.^25^

Despite the numerous benefits of hip arthroscopy, the repair of the acetabular labrum is highly specialized and associated with a challenging learning curve.^26^ The procedure of ALD is generally less challenging than that of ALR for lower-volume surgeons, and the surgeons who performed labral debridement initially may be more likely to investigate failures of treatment or complications than those who performed repairs. On the other hand, it is possible that labral repairs were pursued selectively by more experienced and higher-volume surgeons, leading to biased reporting of outcomes.^27^ The included studies used a variety of clinical and radiographic outcome measures. Finally, the included studies generally had a follow-up period of less than 3 years; hence, the long-term outcome of labral management remains unknown.

### Limitations

This systematic review is not without its limitations. First, the majority of included studies were nonrandomized and comparative. This may increase the risk of selection bias. Forest Plots were used to compare the 2 groups with patient-reported outcomes as an endpoint. Although asking the same question, this faces limitations because the studies analyzed were retrospective and therefore subject to inherent bias. Power analyses were not conducted for the studies that did not report significance and therefore may be underpowered to demonstrate what the other studies report. Therefore conclusions interpreted from these plots must be interpreted with caution. Last, because this is a systematic review, it will contain all the limitations of the studies within it. There are multiple confounding factors when interpreting the data in each study, for example the varying length of follow-up, capsular closure, potentially increased surgeon experience, and technology of newer data versus more preliminary studies.

## Conclusion

Current literature suggests that acetabular labral repair may result in superior patient-reported outcomes. However, there appears to be no significant difference in the rate of progression to total hip arthroplasty at up to 10-year follow-up.

## Supplementary Data

ICMJE author disclosure forms
